# Community health care workers’ risk perception of occupational exposure to HIV in Ibadan, south-west Nigeria

**DOI:** 10.4102/phcfm.v4i1.338

**Published:** 2012-07-23

**Authors:** Adamo A. Akinboro, Olabisi P. Adejumo, Comfort A. Onibokun, Eunice A. Olowokere

**Affiliations:** 1 Medical Unit, International Institute of Tropical Agriculture, Nigeria; 2 Department of Nursing, University of Ibadan, Nigeria; 3 Department of Nursing Science, Obafemi Awolowo University, Nigeria

## Abstract

**Background:**

The ultimate goal of all healthcare workers (HCWs) is to maintain a healthy population and environment, and to adequately manage any condition that might constitute a menace to the health of the population at large. However, the central problem arising from the perception of risk of occupationally transmitted Human Immunodeficiency Virus (HIV) infection amongst HCWs is that it may distract them from giving quality healthcare.

**Objectives:**

The study aimed at addressing the following objectives: to explore the effects of level or years of experience on risk perception regarding occupational exposure to HIV, to assess HCWs’ daily work routines’ consistency in accordance with universal precaution guidelines, to determine HCWs’ perception of workplace safety climate and identify factors that may constitute constraints to HCWs in adhering to universal precaution measures.

**Method:**

A descriptive cross-sectional survey design was utilised, using a triangulation method of data collection which involved the use of a questionnaire and direct observational methods using checklists. A total of 143 HCWs were purposively selected to form the study population.

**Results:**

The study showed a very high risk-perception amongst HCWs regarding occupational exposure to HIV and AIDS but a poor compliance with universal precautions in their professional duties. HCWs perception of risk and workplace safety climate did not influence their compliance with universal precautions (Pr > F = 0.2566; Pr > F = 0.2776).

**Conclusion:**

The need for policy guidelines to manage all aspects of risk-perception and practices of HCWs regarding HIV and AIDS in the healthcare settings most especially at the primary health care level need to be considered.

## Abstrait

**La perception du risque d'exposition professionnelle au VIH chez les agents de santé communautaires à Ibadan, dans le Sud-ouest du Nigeria**

**Présentation:**

L'objectif final de tous les agents de santé communautaires (ASC) est de préserver la santé de la population et un environnement sain, et de gérer adéquatement toute maladie susceptible de constituer une menace pour la population dans son ensemble. Cependant, le principal problème découlant de la perception du risque d'infection du VIH transmis sur le lieu de travail chez les ASC est que celle-ci pourrait les détourner de la prestation de soins de santé de qualité.

**Objectifs:**

L’étude avait les objectifs suivants: étudier les effets du niveau ou des années d'expérience sur la perception du risque en termes d'exposition professionnelle au VIH, évaluer la cohérence des routines quotidiennes des ASC au travail conformément aux directives universelles de précaution, déterminer la perception du climat de sécurité au travail des ASC et identifier les facteurs pouvant constituer des contraintes aux mesures de précaution universelle pour les ASC.

**Méthode:**

Une étude transversale descriptive a été utilisée, s'appuyant sur une méthode de collecte de données par triangulation impliquant l'utilisation d'un questionnaire et des méthodes d'observation directes à l'aide de listes de vérification. Au total, 143 ASC ont été sélectionnés pour former la population de l’étude.

**Résultats:**

L’étude a montré une très forte perception du risque d'exposition professionnelle au VIH/Sida chez les ASC, mais un mauvais respect des précautions universelles dans l'accomplissement de leur travail. La perception du risque et du climat de la sécurité au travail n'influençait pas leur respect des précautions universelles (Pr > F = 0.2566; Pr > F = 0.2776).

**Conclusion:**

La nécessité de directives politiques visant à gérer tous les aspects de la perception du risque et les pratiques des ASC concernant le VIH/Sida dans les environnements de soins de santé, et en particulier au niveau des soins de santé primaires, doit être envisagée.

## Introduction

### Setting

Every year, at least half a million people contract Human Immunodeficiency Virus (HIV) in healthcare settings through unsafe practices and the violation of core aspects related to patients’ right to health.^1^ A central tenet of the right to health is that healthcare must be safe. Regardless of the level of development of patients’ countries, people everywhere in the world have the right to a health system that improves their health status, not one that creates grave risks to their health.

Of 35 million healthcare workers (HCWs) worldwide, approximately 3 million experience percutaneous exposure to blood-borne viruses (e.g. hepatitis B, hepatitis C and HIV) each year.^2^ More than 90% of these infections are occurring in low-income countries like Nigeria and Mozambique, and most are preventable.^3^ HCWs have consistently been a target for studies involving the spread of infectious diseases such as HIV and AIDS;^4^ such studies have tended to concentrate on exposure to risk at work, knowledge and attitudes about infectious diseases, and training and practices in the management of these diseases.^5, 6, 7, 8^

HCWs who have occupational exposure to blood are at an increased risk for acquiring blood-borne infections.^9^ The level of risk depends on the number of patients with that infection in the healthcare facility and the precautions the HCWs observe whilst dealing with these patients.

HCWs in sub-Saharan Africa reportedly perceive themselves as being at a higher risk of HIV infection.^5, 10, 11^ This perception of high risk has been linked to a high rate of infection with HIV in the larger population.^12^ Protection of healthcare personnel and patients from HIV transmission may be a bit difficult in some health care settings; for example, from the researchers’ observations during the conduct of this study, protection of health care providers may be challenging in a setting wherein caregivers are allowed to use only two pairs of gloves per day and needles are re-used after a token wash.

HCWs form a large and growing workforce facing unique occupational hazards, such as exposure to human blood and body fluids, which puts them at risk of contracting numerous blood borne infections (BBIs) including the Hepatitis B Virus (HBV), the Hepatitis C Virus (HCV), and HIV.^13, 14^ Studies have documented that the risk of nosocomial transmission of HBV and HCV following needle stick injury is between 6% – 30%^13^ and 3% – 10%^14^ respectively in susceptible HCWs who were not adequately vaccinated and did not receive post-exposure prophylaxis. In the case of HIV, a rate of less than 0.3%^12^ has been reported; however, globally about a thousand HIV infections could occur annually amongst HCWs mostly in developing nations.

HCWs and patients in low income countries are placed at increased risk of infections because of comparatively common unsafe practices. The risk of occupational infections in such countries is worsened by a range of factors including but not limited to hospital overcrowding, lower HCWs’ patient rations, inadequate or unavailability of basic safety equipment, partial awareness of blood and body fluid exposure risk, and the recycling or reuse of contaminated needles and sharp instruments.^12^

The use of personal protective equipment (PPE), adherence to universal precautions, effective post-exposure management, engineered safer devices, injury surveillance, and relevant legislations are amongst strategies which are designed to maximise the safety of care providers and patients in healthcare setting. In many low-income countries, especially sub-Saharan Africa where more than 70% of the global HIV-infected population live^15^, most of these interventions which have been employed in developed countries are rarely available.^4^

As the awareness of risk of HIV infection increases amongst HCWs, concerns that are reasonable sometimes get magnified which leads to inflated fears and exaggerated perceptions of HIV and AIDS related risks. Whilst those over-blown perceptions affect the quality of care given to patients, it can also lead to stigmatisation and discrimination against people living with HIV or AIDS. The estimated actual risk of occupational transmission of HIV in healthcare settings is low (0.3%)^12^, yet the perception of risk appears significant amongst some care providers. With this in mind, the need for studies targeting hospitals and healthcare settings in Nigeria cannot be over-emphasised.

This study amongst HCWs explored the risk perception and practice of community HCWs regarding occupational exposure to HIV, as a basis for providing recommendations for developing educational programmes that will improve HCWs’ practice and safety irrespective of the work climate environment.

#### Objectives

Infection control practices are poor and widespread with a higher prevalence of HIV in Africa. Throughout the developing world, healthcare providers, health facility staff, patients, and the community at large are placed at risk of contracting HIV because of a lack of supplies, poor training, poor awareness about the danger of unsafe infection control practices, and a lack of incentive to observe good practices. Implementing universal precautions has been a major challenge for health care providers. HCWs’ risk perception and practices regarding universal precautions have implications on the transmission and management of HIV and other blood born diseases in both health facilities and within the community. From the foregoing, there is a gap between the realities of HIV and/or AIDS and occupational practices of HCWs in this part of the world. This study attempts to bridge this gap by exploring the following objectives:

- determine the perception of work-related risks or hazards amongst HCWs concerning HIV
- explore the effect of level or length of experience on risk perception regarding occupational exposure to HIV
- assess HCWs’ daily work routines’ consistency with universal precaution guidelines
- assess HCWs’ perception of workplace safety climate.

#### Contribution to the field

This study brought to the fore the constraints that HCWs faced in adhering to the gold standard of universal precaution measures. This study has also provided information that can be utilised by hospital managers to facilitate safe work environment for healthcare services consumers. This can be done using the findings of the study as a premise on which educational programmes of HCWs will be based with a view to ensuring that HCWs are adequately educated and well-sensitised in terms of occupationally-acquired diseases and their prevention.

Health management authorities and officials and HCWs can use findings from this study as a basis for requesting for resources from appropriate authorities, governmental and nongovernmental or donor agencies to meet the resources and funding needs of health centres, and by extension, other health institutions.

The ultimate outcome of this study will be a better-informed and safety-conscious healthcare workforce with a better disposition to utilise personal protective equipments (PPE) in the face of personal, institutional and occupational realities and challenges which cause them to unduly expose themselves and their patients to various debilitating, disabling, and life-threatening diseases. The study has also provided a starting point for other studies in similar settings thereby increasing the existing knowledge-base on risk-perception and practices of HCWs concerning occupational exposure to HIV.

## Ethical considerations

Permission was obtained from the requisite authorities before commencing the study. Participation was voluntary and written informed consent of prospective participants was obtained. Participants were not required to put their names on the questionnaire and responses were treated with confidentiality.

## Reliability and validity of instruments

The reliability of the instrument was determined through the Test-Re-test Method using a similar setting in a nearby Local Government Area (LGA). Pearson's correlation coefficient was used to measure the degree of correlation and it showed a correlation coefficient of 0.7–1.0, thus the instrument was judged reliable.

## Method

### Materials

The instruments for this study were check listed for direct observation and a self-administered questionnaire which was developed from an extensive literature review. Parts of the self-administered questionnaire were also adapted from existing questionnaires used in the surveys of occupational exposure to blood and the risk of blood borne viral infection amongst HCWs in rural Indian.

### Setting

Ibadan is one of the largest indigenous metropolitan areas in sub-Saharan Africa with an estimated population of 2 million inhabitants (according to the 2006 Nigeria national census statistics); people from all parts of Nigeria and other parts of the world are represented in Ibadan. Ibadan metropolis used to be under one local government, namely the Ibadan Municipal Government. The area used to be split into five distinct local government areas (LGAs) in 1991. The five LGAs include the South-east, South-west, North-east, North-west and North-central areas. The study was conducted in Ibadan South-East Local Government Area of Oyo State, South-west Nigeria. The 2006 Nigeria National Population Census put the population of the study setting at 266 046 inhabitants. Ibadan South-east LGA with headquarters in Mapo Hill is the cradle of the LGA in Ibadanland. The inhabitants of the LGA are predominantly Yoruba; however, people of other races and cultures live and work there. The Ibadan South-east LGA is characterised by high population density, inadequate social amenities and services, such as:

- inadequate health and educational facilities
- crowded residences
- poor sanitation at individual and community levels
- inadequate and inaccessible road networks
- lack of potable water
- an erratic electricity supply.

There are eight primary health centres (PHCs) in the LGA, which are staffed by 187 clinical personnel members, including one medical officer, 39 nurses and/or midwives, 76 community health extension workers (CHEWs), 22 community health officers, 41 health assistants, six pharmacy technicians, and eight others making up the laboratory technicians and medical records assistants. There are private hospitals and missionary hospitals in the LGA as well.

### Design

Triangulation methods were employed for the purpose of data collection which includes the use of a self-reporting questionnaire and direct field observation.

### Procedure

The LGA used for the study was purposively selected out of the five LGAs that make up Ibadan municipality due to its high population density with attendant dearth of social infrastructures, poor sanitation, epileptic power supply, scarcity of potable water, and poor road network. The LGA primary health centre is also at the centre of the town and highly patronised by the local inhabitants.

### Analysing

Analysis of data was done with SAS version 9.2; results obtained were plotted on frequency distribution. Cross tabulation was used to examine the relationships occurring between variables. Associations were tested using Analysis of Variance (ANOVA), Chi-square, and regression analysis.

**TABLE 1:** Respondents’ risk-perception regarding HIV and AIDS.

Variables	Degree of risk perception									

	Strongly disagree		Disagree		Undecided		Agree		Strongly agree	
				
	*n*	%	*n*	%	*n*	%	*n*	%	*n*	%
I think about HIV and/or AIDS as a public health problem all the time.	08	5.6	20	14.0	27	19.0	49	34.5	38	26.8
I think I am at risk of contracting HIV as a result of my job.	12	8.5	23	16.2	11	7.8	72	50.7	24	16.9
My risk of contracting HIV is very high.	19	13.4	32	22.5	12	8.5	58	40.9	21	14.8
My risk of contracting HIV is larger than that of other professionals outside healthcare industry.	19	13.4	20	14.1	17	12.0	67	47.2	19	13.4
Unavailability of personal protective equipment such as gloves, gowns, masks, nylon aprons, et cetera, at some times when caring for patients put me at a very high risk.	7	4.9	18	12.7	9	6.3	68	47.9	40	28.2
Not knowing the HIV status of patients before giving them care puts me at very high risk of contracting HIV.	5	3.52	15	10.6	8	5.6	84	59.2	30	21.1
I am highly worried about contracting HIV in the course of my occupation and/or duties.	10	7.0	34	23.9	16	11.3	65	45.8	17	12.0
I am highly worried about the lack of cure and vaccine for HIV and/or AIDS.	8	5.6	32	22.5	11	7.8	59	41.6	32	22.5
Working with a colleague who does not adhere to universal precautions in the course of duty puts me at a very high risk of HIV infection.	8	5.6	40	28.2	21	14.8	49	34.5	24	16.9
An HIV-infected healthcare worker constitutes a very high risk to their colleagues and patients.	18	12.7	30	21.1	9	6.3	58	40.9	27	19.0

## Results

The sample size consisted of 142 HCWs which represented 70% of the health workers in the LGA. The majority of the participants were female (85.9%) with an average age of 35.5 years and a standard deviation of 8.11. The average number of years’ experience was 11 years with a standard deviation of 8.25. Categories of health care providers who participated in the study showed that 37.2% were Community Health Extension Workers (CHEWs), 22.5% were registered nurses and midwives, 24.6% health assistants, and 12.6% were community health officers (CHO).

### Risk-perception regarding HIV and AIDS

Sixty one per cent (61%) of the participants regard HIV and AIDS as a public health issue and two-thirds (*n* = 96) believed that they were at risk of contracting HIV whilst working. More than half of the participants (60.6%) agreed that they stood a higher risk of contracting HIV than non-health professionals. More than half of the respondents posited that their risk of contracting HIV is very high. The study also revealed that 76.1% (*n* = 108) of the participants agreed that non- availability of personal protective equipment (PPE) at some times when caring for patients put them at risk. Eighty per cent (*n* = 114) of the study population claimed that the fact that the HIV status of most patients were not known prior to giving them care put them at a very high risk.

The fact that no vaccine and/or cure for HIV and AIDS are available was a source of worry for 64% of participants. Data from the study also revealed that whilst 51.4% (*n* = 73) of the participants believed that working with a colleague who does not adhere to universal precautions in their work put them at a very high risk, 59.9% agreed that an HIV-infected HCW poses a very high risk to his or her colleagues and patients.

In this study, however, the length of experience of HCWs did not significantly influence their risk perception regarding occupational exposure to HIV and AIDS (*p* = 0.3352)(Table 2).

**TABLE 2:** Experience and risk-perception regarding occupational exposure to HIV.

Source	Degree of freedom	Sum of squares	Mean square	*F*-value	Pr > *F*
Model	1	44.500350	44.500350	0.94	0.3352
Error	140	6662.577114	47.589837	-	-

Total	141	6707.077465	-	-	-

*Source*: Secondary data

#### Perceptions surrounding workplace safety

The findings of this study revealed a generally good perception of workplace safety amongst participants (Table 3). The majority (89.4%) of them (*n* = 127) agreed that the management of their facilities cared about the HCWs’ safety at work and that protection from exposure (85.9%; *n* = 122) to BBIs was a high priority. Nearly all participants (85%; *n* = 135) claimed that staff in their centre always use universal precautions to protect themselves.

**TABLE 3:** Respondents’ perception of workplace safety climate.

Issue	Yes		No		Not sure	
			
	*n*	%	*n*	%	*n*	%
The management in this health centre cares about my safety at work.	127	89.4	11	7.7	4	2.8
Protection of staff from exposure to HIV and other blood borne viruses is of high priority with management in this health centre.	122	85.9	15	10.6	5	3.5
The staff in this health centre always uses the recommended precautions to protect themselves from contact with patients’ blood and body fluids.	135	95.1	4	2.8	3	2.1
The staff in this health centre has had adequate training on how to protect themselves from infection with HIV.	130	91.6	9	6.3	3	2.1
In this health centre, personal protective equipments for staff are always provided when needed.	116	81.7	17	12.0	9	6.3
In this health centre, supervisors and unit heads are very strict about following recommended precautions.	123	86.6	11	7.8	8	5.6
Our health centre is well staffed.	127	89.4	12	8.5	3	2.1
In this health centre, it is easy to discuss work-related problems with senior staff.	120	84.5	18	12.7	4	2.8
My work area is messy in terms of cleanliness.	60	42.3	78	54.9	4	2.8
I have a clear understanding of post-exposure prophylaxis for HIV.	63	44.4	52	36.6	27	19.0
Personal protective equipments for HIV are available in this health centre.	59	41.6	65	45.8	18	12.7
There are no constraints in terms of my ability to protect myself and my patients from infection and/or transmission of HIV in this health centre.	110	79.1	17	12.2	12	8.6

*Source*: Response to study questionnaire

*n*, Given as number of Respondents’.

**TABLE 4:** Respondents’ practices to universal precautions.

Practices of universal precautions	Never		Rarely		Sometimes		Often		Always	
					
	*n*	%	*n*	%	*n*	%	*n*	%	*n*	%
I protect myself against blood and body fluids of all patients regardless of their diagnosis.	8	5.6	1	0.7	5	3.5	19	13.4	109	76.8
I put used needles and other sharp objects into the designated ‘sharps’ containers.	11	7.8	2	1.4	2	1.4	10	7.0	117	82.4
I wear gloves whenever there is a possibility of exposure to blood or other body fluids.	1	0.7	0	-	1	0.7	12	8.5	128	90.1
I wash my hands after removing disposable gloves and after every procedure.	0	-	1	0.7	3	2.1	10	7.0	128	90.1
I wear a waterproof apron whenever there is a possibility of blood or other body fluids splashing on my clothes.	8	5.6	17	12.0	20	14.1	20	14.1	77	54.2
I wear eye protection (i.e. goggles or glasses) whenever there is a possibility of blood or other body fluids splashing on my face.	49	34.5	11	7.8	17	12.0	21	14.8	44	31.0
I do not recap needles that have been contaminated with blood or body fluids.	38	26.8	4	2.8	10	7.0	11	7.8	79	55.6
I promptly wipe up all blood spills and other body fluids with disinfectants.	7	4.9	2	1.4	5	3.5	11	7.8	117	82.4
I cover my broken skin before coming to work.	3	2.1	2	1.4	5	3.5	15	10.6	117	82.4
I report needle stick injury when it occurs by recording it in a book.	25	17.6	20	14.1	4	2.8	14	9.9	79	55.6

*Source*: Responses to study questionnaire

*n*, Given as number of Respondents’.

One hundred and thirty participants (91.6%) believed that staff in their facilities have received adequate training on how to protect themselves; however, less than half (44.4%; *n* = 63) affirmed that they have a clear understanding of post-exposure prophylaxis (PEP) and 59 participants (41.6%) claimed that PEP was available in their health centres.

Data from the study revealed that 81.7% of the participants (*n* = 116) claimed that PEP for staff were always provided when needed and 86.6% (*n* = 123) posited supervisors or unit heads when giving care. Sixty participants (42%) claimed that their work area was messy, 127 participants (89.4%) believed that their health centre was adequately staffed, and 79.1% claimed that there were no constraints to their ability to protect themselves against exposure to BBIs. Only 12.2% (*n* = 17) believed there were some constraints, amongst which 64.7% of the participants (*n* = 11) identified a lack of necessary materials; 41.2% of the participants (*n* = 7) recognised a lack of knowledge of what to do at times as another constraint. Other constraints identified by participants include discomfort in terms of wearing PEP (29.4%; *n* = 5), pressures associated with work (35.3%; *n* = 6), and emergency situations (5.8%; *n* = 1).

From the investigators’ direct field of observation, however, many staff members in the study centres did not adhere to universal procedures (UP) on many occasions. PEPs were scarce and when available, they were rarely used by staff members including the supervisors. Cleanliness of work areas could be rated as average but there were particular instances where very old beds and dirty linen were found in the labour and post-natal wards. The floor of the labour room was dirty with visible stains of blood and amniotic fluids. None of the centres had PEP and neither were there any clear-cut post exposure management protocols in place contrary to the claim of 41.6% (*n* = 59) of the participants.

#### Practice of universal precaution

Data gathered through the self-reporting questionnaire revealed good compliance with UP amongst the HCWs surveyed; 90.2% participants (*n* = 120) claimed that they protected themselves against contact with BBFs of all patients regardless of their diagnosis. Almost all of the participants (98.6%) claimed that they always wear gloves whenever the possibility of exposure to BBFs existed whilst 97 participants (*n* = 68.3) claimed they wore water proof aprons whenever there is a possibility of BBFs splashing on their body or face. One hundred and thirty five (135) participants (93.0%) claimed that they always covered their broken skin-wounds before going to work. Sixty three per cent of the participants used designated sharp containers and 63.4% of the participants reported that they do not recap used and contaminated needles.

Almost all of the participants (97.1%; *n* = 138) claimed that they usually washed their hands before wearing and after removing disposable gloves for every procedure. However, the direct field observations of investigators revealed a sharp contrast to many of the issues raised regarding the adherence to universal precautions. In all the study sites, there were no containers specifically designed for keeping used needles and sharps were disposed off just like any other hospital waste. Contaminated needles were recapped almost all the time. In addition, the investigation discovered that 4 out of the 8 study sites actually had injury log books, and at the centres where the log books were available, they were rarely used when injuries were sustained.

Hand washing was also found not to be properly practiced in all the centres. In some cases, wash hand basin or hand washing facilities were situated far away from the practice areas and staff only washed their hands after many procedures carried out on many patients. Waterproof aprons were found to be available in 5 out of the 8 centres. Even where aprons were available, staff sometimes did not use them. There was no single protective eyewear in any of the study centres.

It was also detected during the field observation that hand gloves were not worn for many of the procedures, for example giving injections, wound dressing, incision and drainages of abscesses, male child circumcision, et cetera., wiping off of spills of blood and body fluids were sometimes delayed and on many occasions, disinfectants were not used, and when used, the concentration was too low to be effective.

Findings from the study revealed that 137 respondents representing 96.5% reported having moderate to high risk perception regarding exposure to HIV whereas 70% of the respondents reported safe practice of universal precautions. Age, length of experience, risk perception and perception of workplace safety were not found to have influence on HCWs adherence to UP but gender was found to influence risk perception; female HCWs have higher risk perception than their male counterpart (Pr = 0.0014 at 0.95 CI).

## Discussion

This study investigated HCWs’ perception and practices regarding occupational exposure to HIV. Majority of HCWs who participated in the study perceived themselves to be at very high risk of HIV and AIDS as a result of occupational exposure. Data revealed a generally high risk-perception regarding occupational exposure to HIV, as 96.48% reported moderate to high risk-perception. This study corroborates the findings of many studies in sub-Saharan Africa, which have severally documented high risk-perception amongst HCWs.^5, 10, 11, 13, 15^. This high perception of risk cannot be unconnected with the high rate of infection with HIV in the larger population.^5^

In addition, HCWs were not significantly different in terms of HIV and AIDS risk perception considering their length of experience (Pr >F = 0.3352). A disturbing finding of the study was an unacceptably low level of compliance with universal precautions amongst HCWs in the discharge of their clinical duties

The promotion of workplace safety is probably an effective way to achieve greater compliance with universal precautions.^12^ Data from this study showed a contrast to this as no significant relationship was established between perception of workplace safety climate and compliance with universal precautions.

The investigators were also able to establish the fact that none of the centres surveyed had PEP available, despite the claim of about 41% of the respondents. Unavailability of PEP treatments have also been established in hospitals in Kenya and many countries in sub-Saharan Africa.

Occupational exposure to HIV and other Blood Borne Virus (BBV) is unnecessarily common. Regular exposures is as a result of a failure to follow recommended procedures, including the safe handling and disposal of needles and syringes, or wearing personal protective eyewear where indicated. The non-availability of PEP in all the health centres surveyed and possibly beyond is a highly disturbing discovery considering the number of patients the HCWs attend to daily, the prevalence of poor compliance with universal precautions and in the face of growing HIV prevalence in the larger population. It is therefore pertinent that governments and policymakers should not only ensure HCWs have adequate training on PEP, but PEP should be constantly available in all centres considering the fact that accidents do happen even when all the necessary precautionary measures have been taken.

Another interesting finding from this study is that the majority of the HCWs surveyed (79%) claimed that there were no constraints to their ability to protect themselves from exposure to HIV despite the observable evidences of lack of major safety equipments in some health care settings. This finding is quite doubtful, and more extensive research will be necessary to substantiate this. However, constraints identified by few of the participants (12.2%) overlap with those reported in studies amongst American and Thai nurses.^17, 18, 19^

**TABLE 5:** Respondents’ levels of risk perception.

Risk-perception level			Cumulative	
			
	*f*	%	*f*	%
High	93	65.49	93	65.49
Moderate	44	30.99	137	96.48
Low	5	3.52	142	100.00

*Source*: Secondary data

*f*, Frequency.

**TABLE 6:** Respondents’ categorisation of practice and/or adherence to universal precautions.

Universal precautions			Cumulative	
			
	*f*	%	*f*	%
Safe practice	99	69.72	99	69.72
Unsafe practice	43	30.28	142	100.00

*Source*: Response to study questionnaire

*f*, Frequency.

Universal precautions have been in place since 1987^20^ to protect HCWs as well as to prevent HIV and other infectious diseases from being transmitted from HCWs to their patients. However, findings from this study however demonstrated a very low level of compliance with UP amongst community members because only 31% reported overall compliance with all the items on the compliance scale on the instrument. Although 70% of the respondents reported good compliance with UP in the course of their duties, findings from the researcher's direct field observation revealed an unacceptably poor level of compliance with UP by HCWs in the study settings. This is in agreement with several studies globally where sub optimal adherence or a poor level of compliance has been documented extensively, despite evidence that failure to use barrier precautions increases the risk of mucocutaneous blood and body fluids exposure and adherence decreases risk.^12, 17, 18, 19, 21, 22, 23, 24, 25^

Despite sharp contrasts between health systems in America and Nigeria, a sub optimal compliance with UP was noted amongst HCWs in both settings.^24^ If the level of compliance with UP is described as sub optimal in America where health facilities supposedly function efficiently and maximally, supplies and PPEs are always available, training of personnel is a constant and there is a very strict infection control protocol with care audit, it will only be fair to describe the level of practice in Nigeria as disturbing considering the abysmally poor infrastructure, poor funding of healthcare, lack of training amongst many other factors. A critical issue becomes pertinent considering these findings. Workplace environment, training of HCWs, availability of supplies and good functional infrastructure not only determines compliance. Other determinant factors are present and should be elicited by further studies for appropriate intervention.

The study revealed some of the challenges faced by HCWs regarding occupational risks and/or hazards during the performance of their professional duties. The qualitative and quantitative approaches employed in data gathering brought to the limelight the real practices of HCWs *vis-à-vis* the application of universal precaution measures at all times during their routine clinical duties. The study also brought forward constraints of HCWs in adhering to the gold standard, which includes universal precaution measures. Findings from the study will help authorities to plan and strategise in order to ensure that all constraints are addressed in an attempt to facilitate a safe work environment for HCWs and safe health institutions for the healthcare service consumers.

Extrapolations from this study will re-awaken the consciousness of HCWs and their compliance with universal measures as a veritable means of protecting both HCWs and their patients against occupationally transmitted diseases, especially those that are acquired through exposure to blood and body fluids.

Policymakers and invariably HCWs at all levels of care but especially the primary care level can use findings from this study to re-organise, restructure, and repackage healthcare services for the benefit of both the healthcare services providers and the community they serve. In addition, institutions involved in the training of HCWs will also find the result of this study useful in redesigning and or restructuring the educational programmes of HCWs with a view to ensuring students are adequately educated and well-sensitised on occupationally-acquired diseases and their prevention.

Health management authorities and officials and HCWs can use findings from this study as a basis for requesting for resources from appropriate authorities, governmental and non-governmental and/or donor agencies to meet the resources or funding needs of health centres, and by extension, other health institutions.

The ultimate impact of the study will be a better informed and safety-conscious healthcare workforce with a better disposition to utilise personal protective equipment (PPE) in the face of personal, institutional and occupational realities and challenges which cause them to unduly expose themselves and their patients to various debilitating, disabling, and life-threatening diseases.

### Practical implication

The results of this research indicate that protocol and prevention strategies alone are insufficient to ensure a minimal level of risk perception or reduce fear and anxiety about HIV and/or AIDS. Together with increasing the overall awareness about HIV and AIDS issues, remedying the problems of understaffing, inadequate administrative support, poor morale in hospitals, provision of necessary consumables, and PPE and beliefs around occupational exposure to HIV and AIDS are amongst the most important steps in building a safer working environment.

### Limitation of the study

The survey sample was not a probability sample and therefore not representative of all HCWs in Ibadan; therefore, the study findings should be generalised with caution. Another caveat is that by combining health centres, health centre-professional interaction effect is ignored. In addition, social desirability bias could have influenced HCWs responses in a number of areas, including compliance with UP, perception of barriers to safe practice and workplace safety climate.

### Recommendation

Healthy healthcare providers are germane for a strong healthcare system and adherence to universal precautions is highly essential for their safety and well-being. Many countries, particularly in Africa, are facing severe shortages of health professionals and other healthcare providers. Minimising reductions in the healthcare workforce by improving occupational safety through the implementation of UP is an important step in retaining qualified and experienced staff. Governments at all levels and health policy makers should be guided by the perceived barriers to workplace safety so as to fashion ways of addressing them. Some of the perceived barriers will require institutional solutions; for example, inadequate or lack of necessary materials, pressure of work, and lack of requisite knowledge of what to do sometimes.

The management commitment to workplace safety and protection of staff from exposure to HIV and other BBIs was reported in this study. This certainly portends a good management support in healthcare delivery at the PHC levels. Studies will be needed to ascertain whether similar perception of workplace safety climate amongst HCWs permeate other levels of healthcare delivery systems in Nigeria and other parts of Africa.

Even though the study showed that the majority of the respondents had adequate training on how to protect themselves from infection with HIV. This finding calls to question the quality and content of such training in the face of abysmally disappointing levels of adherence with universal precautions, which cuts across all the categories and cadres of HCWs surveyed in the study centres. Again, if they have been adequately trained as they claimed, and they were still found to be grossly noncompliant with standard precautions that are meant to protect them from infection, the possibility of presence of some barriers cannot be ruled out here. As a result, more studies are needed to elicit the perceived barriers to compliance with universal precautions.

The deficient knowledge of respondents about what post exposure prophylaxis (PEP) is all about in the study further reinforced the fact that the claim of adequate training by respondents cannot be sustained, or at least such trainings are grossly deficient. Efforts should be directed at ensuring a comprehensive training, which takes into account HCWs limitations and characteristics. All HCWs in hospital and elsewhere (e.g. general medical and dental practitioners, and community healthcare workers) should be informed and educated about the possible risks from occupational exposure and should be aware of the importance of seeking urgent advice following any needle stick injury or other occupational exposure.

In all the study sites, hospital wastes and sharps were not separated and there were no designated sharp containers in all the centres surveyed. It simply implies that theoretical knowledge of respondents in managing hospital wastes is not translated into practice. Education, training and provision of needed materials at all times may then not be enough to ensure compliance, mechanisms should be put in place to enforce institutional rules and total compliance with UP protocols most especially in the low resource countries,

## Conclusion

In caring for patients with or without HIV and AIDS, HCWs may have ‘misconceptions’ relating to the risk of HIV infection that interferes with their ability to provide quality care. However, they have a moral and ethical responsibility to care for all patients regardless of their diagnosis. In order not to compromise the quality of care given to patients (especially those living with HIV or AIDS) or to deny them the right to good healthcare, educational programmes in healthcare settings should be geared at addressing the real and imaginary causes of heightened risk-perception which looms large in the minds and choices of HCWs worldwide. This means that educational intervention in this area should be evidence based and designed to suit different health care settings as identified by management and policy makers. Nurses and all other health care givers have the professional right to also protect themselves and their patients by adhering to these simple and straight forward techniques of universal precautions.
